# Evaluation of the Effect of Inactivated Transmissible Gastroenteritis Virus Vaccine with Nano Silicon on the Phenotype and Function of Porcine Dendritic Cells

**DOI:** 10.3390/v13112158

**Published:** 2021-10-26

**Authors:** Lanlan Zheng, Fujie Zhao, Jiaxi Ru, Lintao Liu, Zi Wang, Nianxiang Wang, Xiangli Shu, Zhanyong Wei, Huichen Guo

**Affiliations:** 1 College of Veterinary Medicine, Henan Agricultural University, Zhengzhou 450002, China; lanlan@henau.edu.cn (L.Z.); zfj216403@163.com (F.Z.); liulintao1229@163.com (L.L.); wang1998zi@163.com (Z.W.); wangnianxiang2021@163.com (N.W.); xianglishu163@163.com (X.S.); 2 State Key Laboratory of Veterinary Etiological Biology, Lanzhou Veterinary Research Institute, Chinese Academy of Agricultural Sciences, Lanzhou 730046, China; dfbb_3277@163.com

**Keywords:** inactivated TGEV vaccine, nano silicon, dendritic cell, innate immunity

## Abstract

A transmissible gastroenteritis virus (TGEV) is a porcine enteropathogenic coronavirus, causing acute swine enteric disease especially in suckling piglets. Mesoporous silica nanoparticles (MSNs) are safe vaccine adjuvant, which could enhance immune responses. Our previous research confirmed that nano silicon had immune-enhancing effects with inactivated TGEV vaccine. In this study, we further clarified the immune-enhancing mechanism of the inactivated TGEV vaccine with MSNs on porcine dendritic cells (DCs). Our results indicated that the inactivated TGEV vaccine with MSNs strongly enhanced the activation of the DCs. Expressions of TLR3, TLR5, TLR7, TLR9, and TLR10, cytokines IFN-α, IL-1β, IL-6, IL-12, and TNF-α, cytokine receptor CCR-7 of immature DCs were characterized and showed themselves to be significantly higher in the inactivated TGEV vaccine with the MSN group. In summary, the inactivated TGEV vaccine with MSNs has effects on the phenotype and function of porcine DCs, which helps to better understand the immune-enhancing mechanism.

## 1. Introduction

A transmissible gastroenteritis virus (TGEV) is a porcine enteropathogenic coronavirus, which belongs to the family Coronaviridae, and causes acute swine enteric disease with viral enteritis, severe watery diarrhea, vomiting, dehydration, and high mortality rates, especially in suckling piglets [1,2]. TGEV was first reported in 1946 in the United States. Since then, it has spread in swine herds worldwide, causing heavy economic losses in the pig industry around the globe [3,4]. Currently, several kinds of TGEV vaccines have been developed, most of which are administered to the pregnant sows during gestation to provide lactogenic immunity to newborn piglets. However, low levels of protection against TGEV were reported, and we should still pay great attention to this virus [5].

Inactivated vaccines have been widely used due to their high level of safety. However, they usually require the addition of adjuvants to achieve their good immunogenicity. Many kinds of adjuvants are used in animals, such as alum, liposomes, and emulsions [6]. The development of nano adjuvants provides a new way for vaccine enhancement [7]. Nano adjuvants are nano sized materials with strong adsorptive ability, good slow-release function, effective targeting, and thermal stability [8]. Because of these advantages, nanoparticles have been used as adjuvants to improve the immunogenicity of the antigen. In our previous research [9], a kind of nano silicon particle was applied to inactivated TGEV vaccine, and the immune-enhancing effects were investigated in mice. The results showed that nano silicon could be used as an adjuvant for an inactivated TGEV vaccine, enhancing the long term humoral and early cellular immune responses, with high antibody titers, increasing cytokine expression, and CD4^+^/CD8^+^ T lymphocyte proliferation. However, the immune-enhancing mechanism of nano silicon is still not clear.

Nanoparticles could interact with the immune system and internalized by dendritic cells (DCs) [10]. DCs, the most important antigen-presenting cells (APC) of the innate immune system, provide the first line of defense, which could recognize, capture, and internalize antigens with nanoparticles by initiating immune reactions. Upon stimulation, immature DCs (im-DCs) undergo a maturation process into mature DCs (m-DCs), with upregulation of co-stimulatory molecules and maturation markers, activation of their toll-like receptors (TLRs), and then m-DCs migrate to regional lymphoid organs to activate T cell differentiations [11]. Given that DCs are essential for bridging innate and adaptive immune responses [12,13], an in-depth understanding of the phenotypic and functional changes of DCs when exposed to the inactivated TGEV vaccine with nano silicon is essential to clarify the immune-enhancing mechanism. However, the effect of the inactivated TGEV vaccine with nano silicon on ex vivo peripheral blood DCs of porcine has not been reported.

In this study, the effects of the inactivated TGEV vaccine with mesoporous silica nanoparticles (MSNs) on the phenotype and function of porcine DCs were evaluated. Phenotype and function of the DCs were assessed by uptake and intracellular localization studies, identifying expression levels of maturation markers of DCs, TLRs expression, and the stimulatory capacity of cytokines and cytokine receptor. A better understanding of these mechanisms could pave the way for improved vaccination strategies.

## 2. Materials and Methods

### 2.1. Fluorescent Labeling of MSNs

Dendrimer-like MSNs (50 nm) were synthetized by the State Key Laboratory of Veterinary Etiological Biology, Lanzhou Veterinary Research Institute [14]. In addition, 100 mg of MSNs dispersed in 30 mL toluene (Solarbio, Beijing, China), followed by adding 100 μL of aminopropyltriethoxysilane (APTES) (Solarbio, Beijing, China), and the mixture was treated at 110 °C for 20 h. Afterward, the MSNs were washed three times with ethanol and centrifuged at 10,000 rpm for 10 min. One-hundred milligrams of aminated MSNs were then labeled with fluorescein isothiocyanate (FITC) (Sigma, St. Louis, MO, USA). Briefly, MSNs dispersed in 50 mL ethanol with ultrasonic treatment for 10 min, and then 10 mg FITC was added to MSNs. Samples were placed on an orbital shaker in the dark overnight (RT, 60 rpm). MSNs were washed several times with ethanol (10,000 rpm, 10 min) until the supernatant was colorless. After the final wash, MSNs were re-suspended in phosphate buffer saline (PBS) at a final concentration of 1 mg/mL.

### 2.2. Preparation of Inactivated TGEV Vaccine with MSNs

A swine testicle (ST) cell line (CRL-1746; ATCC) was cultured in Dulbecco’s modified Eagle’s medium (DMEM) (Hyclone, Logan, UT, USA) supplemented with 10% fetal bovine serum (FBS) (Gibco, Carlsbad, CA, USA) at 37 °C in a 5% CO_2_ incubator, and was preserved by the Key Laboratory for Animal-Derived Food Safety of Henan Province. TGEV HN-2012 strain was isolated and identified by the Key Laboratory for Animal-Derived Food Safety of Henan Province, and propagated in ST cells. Inactivated TGEV was prepared as described in our previous research [9]. Briefly, the tissue culture infective dose (TCID_50_) was calculated by using the Reed–Muench method, and the virus titer was determined to be 10^8.0^ TCID_50_/0.1 mL. TGEV was then inactivated by binaryethylenimine (BEI) with the final concentration of 0.04% (*w*/*v*) for 12 h [15]. The inactivation of TGEV was confirmed by a lack of infectivity in ST cells. MSNs in PBS were filtered, sterilized, and then mixed with inactivated TGEV in a volume ratio of 1:1, shaking at 4 °C overnight to prepare the vaccine. In addition, the control groups of the inactivated TGEV or the MSNs were also prepared with the same amount as the vaccine. The zeta potential values of the inactivated TGEV vaccine, MSNs, and the complex of the inactivated TGEV vaccine with MSNs were measured by Zetasizer Nano ZSE 3700 (Malvern, Malvern, UK).

### 2.3. Animal Experiments

Three two-month-old female pigs were purchased from a pig farm with a high level of health status in the Henan province of China, and maintained in isolated cages free of a variety of pathogens. Before the experiment, blood and fecal samples of pigs were collected to confirm that the pigs were negative for TGEV.

### 2.4. Peripheral Blood Mononuclear Cells Isolation

Blood was collected from the pigs randomly by a puncture of the anterior vena cava and anticoagulated with citrate, and the peripheral blood mononuclear cells (PBMCs) were isolated by density centrifugation [16]. Briefly, the collected blood was diluted with PBS, and gently added to the same volume of lymphocyte separation medium (Solarbio, Beijing, China). After centrifuged at 1200× *g* for 30 min, the buffy coat was obtained and washed twice with PBS at 300× *g* for 10 min. The PBMCs were re-suspended in RPMI-1640 medium (Hyclone, Logan, UT, USA) supplemented with 10% FBS, 1% penicillin/streptomycin (Gibco, Carlsbad, CA, USA), and counted.

### 2.5. Monocyte Derived DC Culture

PBMCs (4 × 10^6^ cells/mL) were isolated by density centrifugation and cultured in RPMI-1640 medium supplemented with 10% FBS and 1% penicillin/streptomycin at 37 °C for 4 h. The adherent cells were collected and then cultured in RPMI-1640 medium supplemented with 20 ng/mL of porcine granulocyte macrophage colony stimulating factor (GM-CSF) (R&D Systems, Emeryville, CA, USA) and 20 ng/mL of porcine interleukin 4 (IL-4) (R&D Systems, Emeryville, CA, USA) at 37 °C for 5 days. On day 5, im-DCs were induced maturation with 1 μg/mL of lipopolysaccharide (LPS) (Sigma, St. Louis, MO, USA) for 24 h [17]. The DC morphology was examined under a microscope at different times. The cells were aggregated obviously, and dendrites increased gradually.

### 2.6. MSN Toxicity Measure on DCs

Im-DCs (1 × 10^6^ cells/mL) were cultured in 96-well plates, and incubated with increasing concentrations of MSNs to examine whether MSNs were toxic to DCs. MSNs were dissolved and added to im-DCs at the final concentrations of 31.25 μg/mL, 15.63 μg/mL, 7.81 μg/mL, and 3.91 μg/mL, respectively. After incubation at 37 °C for 24 h, the cells were incubated with 10 μL of CCK-8 (Solarbio, Beijing, China). After incubation at 37 °C for 4 h, the viability of DCs was assessed by OD_450_ using an ELISA plate reader (Bio-Tek, Burlington, VT, USA). Cell viability was calculated using the following equation: cell viability (%) = (OD_450_ of the DCs treated with MSNs-OD_450_ of the DMEM)/(OD_450_ of the negative control of DCs-OD_450_ of the DMEM) × 100%.

### 2.7. Study of DC Uptake of MSNs

To analyze the uptake efficiency of DCs, im-DCs (1 × 10^6^ cells/mL) were cultured with FITC-dextran (Sigma, St. Louis, MO, USA) at the final concentration of 1 mg/mL, and 10 μL of 1 mg/mL FITC labeled MSNs (FITC-MSNs), respectively. The cells were then placed at 37 °C for 2 h in the dark. The cells were washed twice with 0.01 M PBS at 2500 r/min for 5 min, and the excess of FITC-dextran or FITC-MSNs was discarded. The cells were assessed by flow cytometry, and the mean fluorescence intensity (MFI) was recorded. At the same time, the im-DCs cultured with FITC-dextran or FITC-MSNs were incubated at 4 °C in the dark as negative controls, and the im-DCs without treatment were set as blank controls. The phagocytic capacity of im-DCs was evaluated by ΔMFI (ΔMFI = the value of MFI under 37 °C treatment—the value of MFI under 4 °C treatment).

### 2.8. Intracellular Localization of MSNs

Im-DCs (1 × 10^6^ cells/mL) cultured with 10 μL of 1 mg/mL MSNs were fixed and embedded in resin for viewing under transmission electron microscopy (TEM) [18]. Briefly, im-DCs were cultured with MSNs at 37 °C for 120 min in the dark, and the cells added with 0.01 M PBS were negative control. The cells were washed twice in 0.01 M PBS at 2500 r/min for 5 min, and the excess of MSNs was discarded. Cells were fixed with 2.5% glutaraldehyde (Servicebio, Wuhan, China) for later sectioning and then assessed by TEM.

### 2.9. Identification of DC Phenotype

Im-DCs (1 × 10^6^ cells/well) were harvested and cultured in 12-well plates. In addition, 10 μL of 1 mg/mL MSNs with 10 μL PBS, 10 μL of 1 mg/mL MSNs with 10 μL inactivated TGEV, 10 μL of inactivated TGEV with 10 μL PBS, 20 μL of 1 μg/mL of LPS, and 20 μL PBS were added to the cells, respectively. Cells were harvested at 24 h and incubated with FITC conjugated anti-porcine major histocompatibility complex (MHC) class II (FITC-MHCII) antibody (Bio-Rad, Hercules, CA, USA) and allophycocyanin (APC) conjugated anti-porcine CD80 (APC-CD80) antibody (Invitrogen, New York, CA, USA) for 20 min at 4 °C in the dark [19]. The samples were stained in triplicate, the activation state of DCs was determined by the analysis of the phenotype using flow cytometry (BD Biosciences, Franklin, NJ, USA), and the data were analyzed using Canto diva (BD Biosciences, Franklin, NJ, USA) software.

### 2.10. Quantifications of TLRs, Cytokines, and Cytokine Receptor Expressions in DCs

Im-DCs (1 × 10^6^ cells/well) were harvested and cultured in 12-well plates. In addition, 10 μL of 1 mg/mL MSNs with 10 μL PBS, 10 μL of 1 mg/mL MSNs with 10 μL inactivated TGEV, 10 μL of inactivated TGEV with 10 μL PBS, and 20 μL PBS were added to the cells, respectively. Cells were harvested after 3 h, 6 h, 12 h, and 24 h, and the mRNAs of TLRs were detected by real-time PCR. The supernatant was harvested and cytokine and cytokine receptor quantifications were performed using a real-time PCR assay. The primers used are shown in Table 1.

### 2.11. Statistical Analysis

Statistical analysis was performed by SPSS version 17.0 software (GraphPad Prism, GraphPad Software, Inc., La Jolla, CA, USA). Duncan’s multiple range tests and chi-square test were used to determine differences among groups. Statistical significance is indicated on the figures.

## 3. Results

### 3.1. Characterization of the Inactivated TGEV Vaccine with MSNs

**Figure 1:**
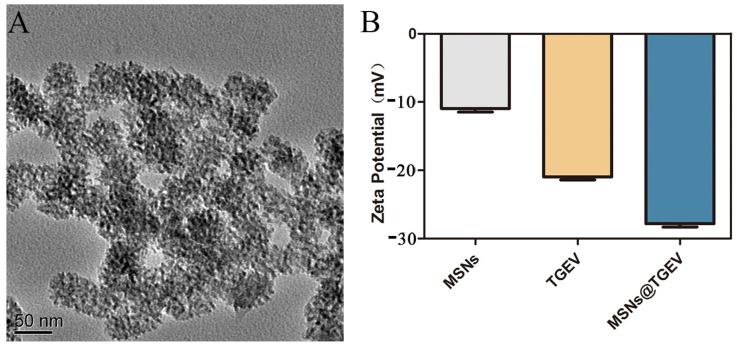
Morphology of MSNs and Zeta potential measurements. (**A**) The uniform spherical of MSNs was confirmed by TEM. (**B**) The complex of inactivated TGEV vaccine with MSNs was investigated by Zeta potential measurement.

The uniform spherical morphology of MSNs was confirmed with a diameter of about 50 nm by TEM (Figure 1A). In addition, the complex of inactivated TGEV vaccine with MSNs had been further investigated by zeta potential measurement (Figure 1B). From the zeta potential measurements, we found that the value of zeta potential of the inactivated TGEV vaccine with MSNs was changed after the complex formed, indicating that the inactivated TGEV was successfully loaded on MSNs.

### 3.2. Toxicity of MSNs on im-DCs

**Figure 2:**
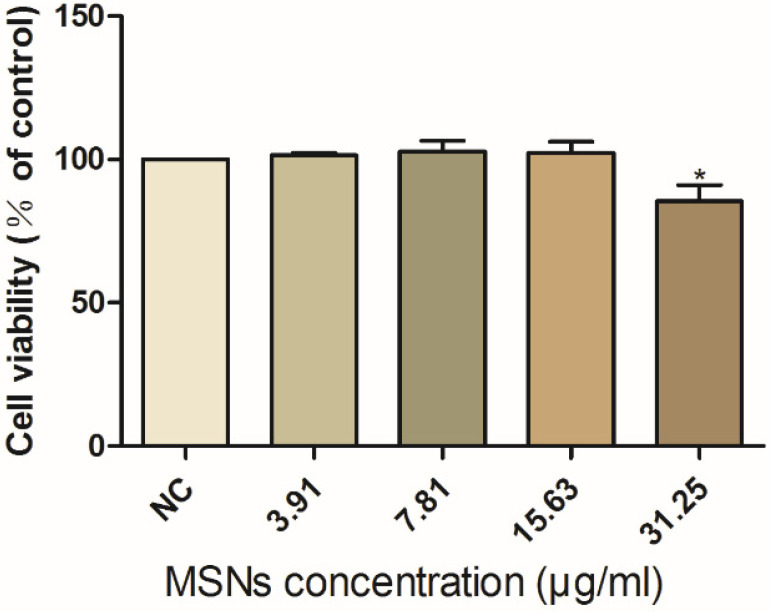
Toxicity detection of MSNs on im-DCs. Different concentrations of MSNs were incubated with im-DCs for 24 h, and the viability of DCs was assessed. Data were presented as mean ± SD of three duplicate samples. Differences were considered significant at (*) *p* < 0.05.

In this study, the toxicity of MSNs to porcine im-DCs was investigated. Firstly, we assessed the status of the im-DCs. After being induced with LPS for 24 h, the DC morphology was examined under a microscope, the cells were aggregated obviously, and dendrites increased gradually. Im-DCs were incubated with increasing concentrations of MSNs for 24 h, and the viability of DCs was assessed. The results demonstrated that MSNs had no toxicity on DCs when the concentration reached up to 15.63 μg/mL (Figure 2).

### 3.3. Uptake and Intracellular Localization of MSNs in im-DCs

**Figure 3:**
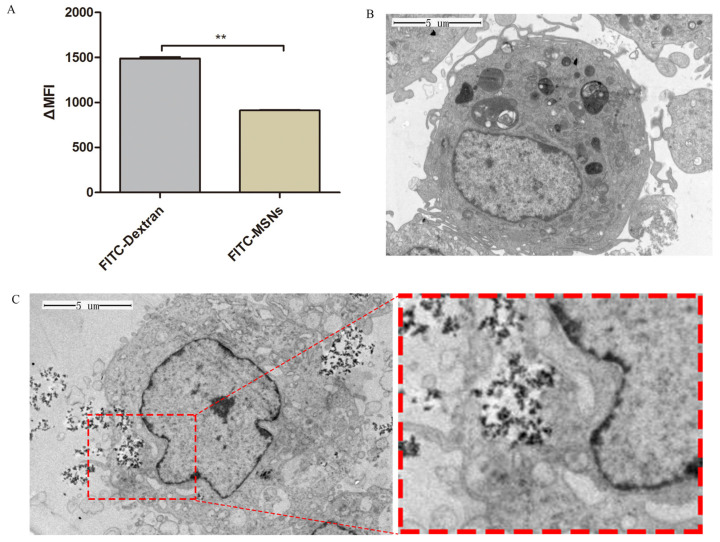
Uptake and intracellular localization of MSNs by im-DCs. (**A**) The phagocytic capacity of im-DCs with FITC-MSNs was detected by flow cytometry. DCs cultured with FITC-dextran as a positive control. MFI were plotted using GraphPad Prism; (**B**) TEM image of DCs untreated as a negative control; (**C**) TEM image of internalization of MSNs in DCs. Data were presented as mean ± SD of three duplicate samples. Differences were considered significant at (**) *p* < 0.01.

To examine whether MSNs were taken up by im-DCs and analyze their uptake efficiency, FITC-MSNs were added to im-DCs as well as analyzed by flow cytometry. As shown in Figure 3A, the strong FITC fluorescence signal could be detected in im-DCs after being placed with FITC-MSNs at 37 °C for 2 h, which concluded that MSNs were taken in im-DCs. The phagocytic capacity of im-DCs was also detected with FITC-dextran by flow cytometry as a positive control.

To specifically determine whether MSNs were localized intra- or extracellular, TEM was used to perform an in-depth analysis of DCs. After im-DCs and MSNs cultured at 37 °C for 2 h, high numbers of MSNs were found inside DCs, confirming that MSNs were effectively taken up by DCs and localized intracellularly. MSNs were bonded and internalized by DC after being cultured for 2 h (Figure 3C), while the structure of the DC containing MSNs appeared normal, although their cytoplasm was rich in MSNs.

### 3.4. Inactivated TGEV Vaccine with MSNs Promoting DC Maturation

**Figure 4:**
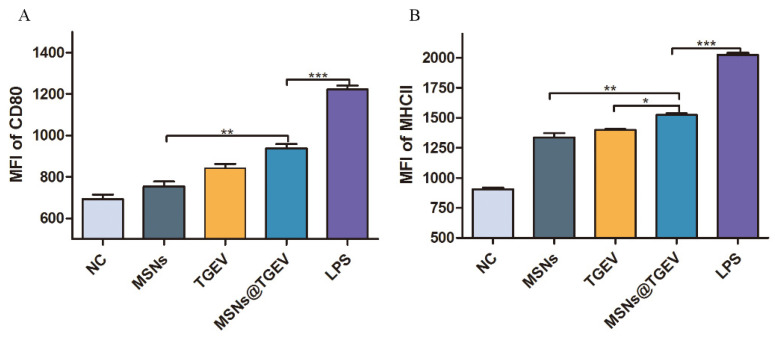
Inactivated TGEV vaccine with MSNs promotes DC maturation in vitro. The im-DCs were treated with MSNs, inactivated TGEV, and inactivated TGEV vaccine with MSNs for 24 h, respectively. The maturation of DCs was investigated by the expression of MHCII and CD80. Untreated im-DCs were negative control, and im-DCs treated with LPS were positive control. MFI were plotted using GraphPad Prism. (**A**) the expressions of CD80; (**B**) the expressions of MHCII. Data were presented as mean ± SD of three duplicate samples. Differences were considered significant at (*) *p* < 0.05, (**) *p* < 0.01, (***) *p* < 0.001.

In order to evaluate the impact of inactivated TGEV vaccine with MSNs on DCs, the maturation of the im-DCs after being stimulated by the inactivated TGEV vaccine with MSNs was investigated by the expressions of the cell-surface molecule MHCII and costimulatory molecules CD80. The results showed that the expressions of MHCII and CD80 were the most prominent in the inactivated TGEV vaccine with the MSN group, except for the LPS positive control group. The lower expression levels were found in the inactivated TGEV group and MSN group (Figure 4). CD80 expression was significantly increased in the inactivated TGEV vaccine with MSN group (Figure 4A). The inactivated TGEV vaccine with MSNs significantly increased MHC-II expression after 24 h of stimulation, in comparison with the inactivated TGEV group (Figure 4B) (*p* < 0.05) or MSNs group (*p* < 0.01).

### 3.5. mRNA Expression of TLRs

**Figure 5:**
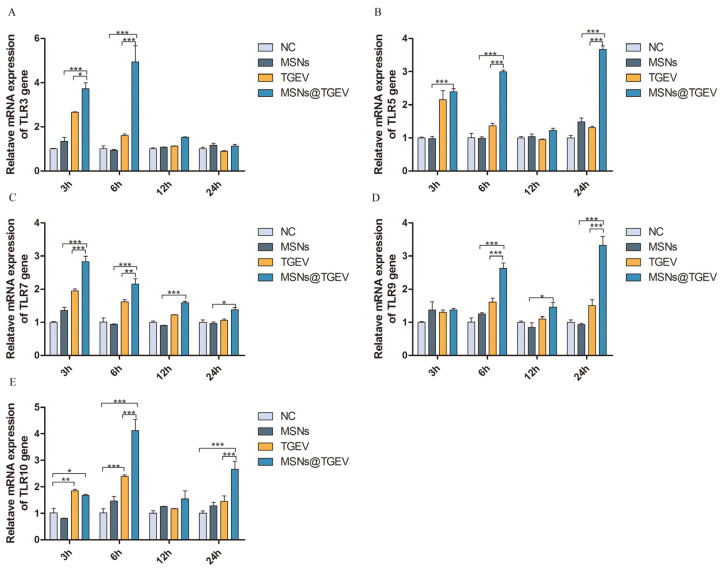
mRNA expression of TLRs on DCs after incubation with the inactivated TGEV vaccine with MSNs for different times. Im-DCs were cultured with MSNs, inactivated TGEV and inactivated TGEV vaccine with MSNs, and cells were harvested at 3 h, 6 h, 12 h, and 24 h, respectively. Im-DCs untreated were negative control. mRNA expressions of TLR1-10 were assessed by real-time PCR. (**A**) the expressions of TLR3; (**B**) the expressions of TLR5; (**C**) the expressions of TLR7; (**D**) the expressions of TLR9; (**E**) the expressions of TLR10. Data were presented as mean ± SD of three duplicate samples. Differences were considered significant at (*) *p* < 0.05, (**) *p* < 0.01, (***) *p* < 0.001.

To investigate if any differences were observed that related to TLR expression, we assessed mRNA expressions of TLR1-10 in the im-DCs when treated with the inactivated TGEV vaccine with MSNs for different times. mRNA expressions of TLR3, TLR5, TLR7, TLR9, and TLR10 showed significant differences among different groups (Figure 5), while TLR1, TLR2, TLR4, TLR6, and TLR8 had no significant differences in all groups at different times (data not shown). Specifically, mRNA expressions of TLR3, TLR5, and TLR7 were significantly higher than that of other groups at 3 h (Figure 5A–C) (*p* < 0.001), and mRNA expression of TLR7 reached the highest at 3 h (Figure 5C), mRNAs expression of TLR3 as well as TLR10 reached their peaks at 6 h (Figure 5A,E), and mRNAs expression of TLR5 and TLR9 were the highest at 24 h (Figure 5B,D).

### 3.6. mRNA Expression of Cytokines and Cytokine Receptor

**Figure 6:**
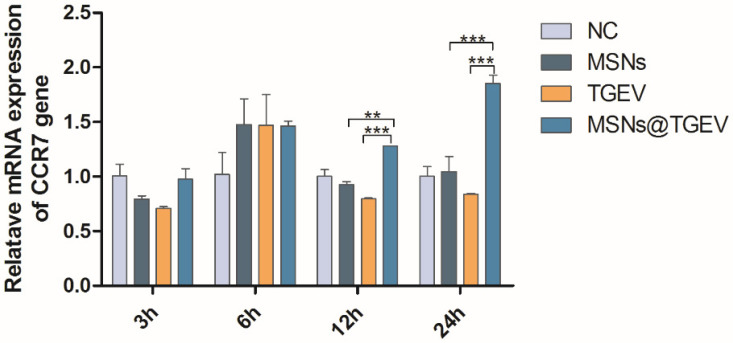
mRNA expression of CCR7 on DCs after incubation with the inactivated TGEV vaccine with MSNs for different times. Im-DCs were cultured with MSNs, inactivated TGEV and inactivated TGEV vaccine with MSNs, and cells were harvested at 3 h, 6 h, 12 h, and 24 h, respectively. Im-DCs untreated were negative control. mRNA expression of CCR7 was assessed by real-time PCR. Data were presented as mean ± SD of three duplicate samples. Differences were considered significant at (**) *p* < 0.01, (***) *p* < 0.001.

**Figure 7:**
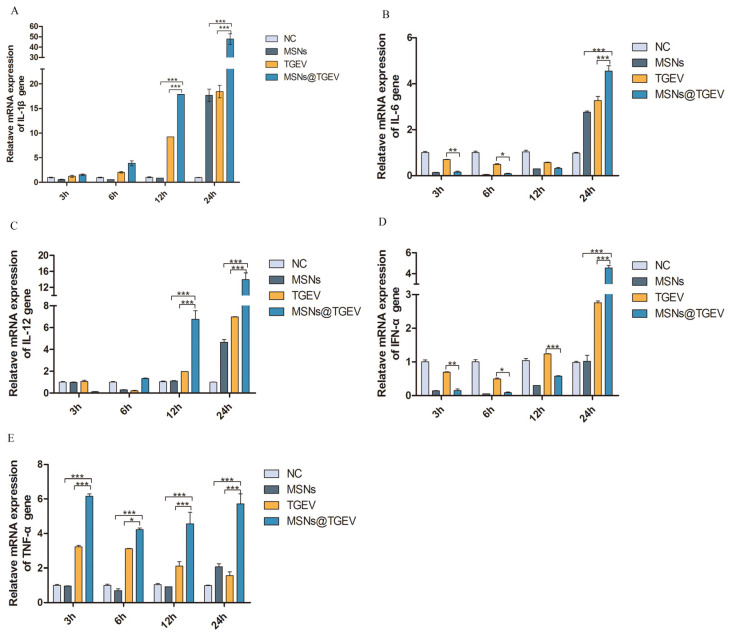
mRNA expression of cytokines on DCs after incubation with inactivated TGEV vac cine with MSNs for different times. Im-DCs were cultured with MSNs, inactivated TGEV and inactivated TGEV vaccine with MSNs, and cells were harvested at 3 h, 6 h, 12 h, and 24 h, respectively. Im-DCs untreated were negative control. mRNA expression of cytokines was assessed by real-time PCR. (**A**) the expressions of IL-1β; (**B**) the expressions of IL-6; (**C**) the expressions of IL-12; (**D**) the expressions of IFN-α; (**E**) the expressions of TNF-α. Data were presented as mean ± SD of three duplicate samples. Differences were considered significant at (*) *p* < 0.05, (**) *p* < 0.01, (***) *p* < 0.001.

Im-DCs were stimulated by the inactivated TGEV vaccine with MSNs for different times, and mRNA levels of cytokine interferon-α (IFN-α), pro-inflammatory cytokines IL-1β, IL-6, IL-12, tumor necrosis factor α (TNF-α) and cytokine receptor 7 (CCR-7), which was implicated in immune response, were characterized by a real-time PCR assay (Figure 6 and Figure 7). The inactivated TGEV vaccine with MSNs significantly increased the secretion of IFN-α, pro-inflammatory cytokines IL-1β, IL-6, IL-12, TNF-α, and CCR-7 in DCs, suggesting a direct impact of the inactivated TGEV vaccine with MSNs in the immune responses. More precisely, the results indicated that stimulation of im-DCs with inactivated TGEV vaccine with MSNs induced secretion of high amounts of TNF-α after 3 h (Figure 7E) (*p* < 0.001). The secretion of IL-1β and IL-12 were significantly increased after 12 h (Figure 7A,C) (*p* < 0.001), and high amounts of cytokines IFN-α and IL-6 were expressed at 24 h (Figure 7B,D) (*p* < 0.001). Additionally, the secretion of CCR-7 reached its peak at 24 h (Figure 6) (*p* < 0.001). The negative control group did not show any immune-activating properties.

## 4. Discussion

In recent years, nanomaterials have attracted extensive attention of researchers because of their advantages of strong adsorption properties, slow-release function, effective targeting, and thermal stability. Many of them have been applied in the biological field as adjuvants of vaccines, and induced effective and persistent immune responses [20]. Otherwise, despite intensive research in the field of nanomaterials, fundamental knowledge in understanding the interactions between nanomaterials and the immune system is still unclear. MSNs are kinds of spherical particles with large surface areas, high loading efficiencies, high pore volume, easily tunable porous structure, and good dispersion in solution [21,22]. Their size and structure can be easily tuned. Compared with traditional vaccine adjuvants, MSNs showed strong antigen adsorption capacity with a lower dosage of use, which could significantly improve the protein loading capacity, especially for large protein molecules [23], and have been used as adjuvant in many vaccines to achieve a sustained immune response [24,25]. Moreover, MSNs have good biocompatibility, biodistribution, and biodegradability with low toxicity, and the breakdown product is silicic acid with related molecules, a nontoxic by-product that can be filtered through the kidney [26,27]. TGEV is a porcine enteropathogenic coronavirus, and causes acute swine enteric disease, especially in suckling piglets. Actually, the immunization of TGEV vaccine is usually administered to pregnant sows, which is the best choice to enhance the maternal immunity level. The main protection mechanism of neonatal piglets is mediated by lactogenic immunity. During lactation, specific antibodies including IgG, IgM, and sIgA are passively transferred to piglets via colostrum and milk [28]. In the present study, we aimed to analyze the effects of inactivated TGEV vaccine with nano-sized MSNs adjuvant on DCs in vitro. In order to systematically analyze the immune-enhancing mechanism of MSNs, we have provided an exhaustive investigation of MSN modulation of DC function and showed that MSNs had affected the expression of MHCII and CD80 of DCs.

DCs are the most potent APC and the key effector of immune responses, acting as a bridge between the innate and adaptive immune systems [29]. DCs have various receptors, making them the first types of professional immune cells to recognize different stimuli and initiate immune reactions. However, after maturation, resident DCs capture antigens, migrating to the lymph nodes where they are able to interact with naïve T cells, producing a number of inflammatory cytokines and other factors, initiating T cell adaptive immune responses and stimulating memory responses [30]. The effect of the inactivated TGEV vaccine with MSNs on DCs activation in vivo needs to be further studied.

Since DCs in the blood are very rare, DCs can be generated in vitro by adding GM-CSF and IL-4 to the medium during monocyte culture. These DCs derived from monocytes present dendritic morphology, uptake foreign antigens, and have the unique ability to activate and regulate T lymphocytes. Im-DCs present a high endocytic activity and low expression of MHCII and co-stimulatory molecules. Stimulation induces DC maturation and activation, which closely influences their function, and results in upregulated expression of MHC and co-stimulatory molecules CD80/86 on their surfaces, and the secretion of pro-inflammatory cytokines, all of which are prerequisites for successful induction of immune responses [31]. In addition, antigen presentation and the expression of chemokines related to the immune response are also affected. In response to antigen stimulation, DCs up-regulated CCR7 gene expression [32]. DCs preferentially capture smaller particles, which could induce CD4^+^ T cell proliferation in draining lymph nodes or reduce antigen degradation by interfering with the lysosomal compartment [33,34].

We analyzed uptake efficiency and intracellular localization of MSNs in im-DCs by flow cytometry and TEM. The uptake of MSNs by DCs was measured by determining FITC fluorescence intensities of MSNs. Then, the intracellular localization of MSNs was confirmed by TEM. As expected, due to the phagocytic nature of im-DCs, uptake of MSNs was observed after being cultured for 2 h, which demonstrated that phagocytic cells have a much higher internalization capacity compared with other cell types [35]. They depend on cell type, size, surface charge, shape, and nanoparticle itself. DC maturation is accompanied by the upregulation of MHC-I/MHC-II, CD80/86, as well as factors involved in cell adhesion. To assess the effects of inactivated TGEV vaccine with MSNs on DCs, we analyzed maturation and phenotype of DCs after inactivated TGEV vaccine with MSN treatment. Our data showed that the treatment induced maturation of DCs, and the expression of MHC II and CD80 was also affected, which was consistent with previous research [36].

In this study, expressions of TLRs, cytokines, and cytokine receptor, which are implicated in immune responses were characterized by real-time PCR assay. Unlike DCs of human and mice [37,38], porcine DCs selectively express TLR3, TLR5, TLR7, TLR9, and TLR10, which were significantly higher in the inactivated TGEV vaccine with the MSN group. TLRs are a family of conserved pattern recognition receptors (PRRs) that recognize microbial pathogens. DCs are activated by recognizing antigens using innate immune receptors (including TLRs) and transport them to T cells in the secondary lymphoid organs [39]. The response of porcine DCs to TLR ligands has been studied previously. In general, the cells respond to a wide range of TLR ligands, including TLR2, TLR3, TLR4, TLR5, TLR7/8, and TLR9, while TLR2/4-mediated responses are generally more robust [40]. It has been reported that the TLR signaling pathway induces the secretion of inflammatory cytokines by activating the transcription factor NF-κB and mitogen-activated protein kinases (MAPKs) [41]. Furthermore, nanoparticles such as MSNs have the right size range, foreignness, and they enter via the same routes as pathogens, making them ideal candidates for being recognized by DCs [30]. mRNA expression of TLR7 reached the highest when the im-DCs cultured with the inactivated TGEV vaccine with MSNs for 3 h, which is consistent with the immune surveillance of TLR7. im-DCs cultured with the inactivated TGEV vaccine with MSNs can cause morphological changes of cells, and induce the significant upregulation of TLR3, TLR5, TLR7, TLR9, and TLR10 mRNA transcription levels in cells. TLR3, TLR5, TLR7, TLR9, and TLR10 may recognize inactivated TGEV vaccine with MSNs, or they are activated by certain ligands and exert immune effects. At present, the relationship between inactivated TGEV vaccine with MSNs and TLRs-mediated innate immune response is still in the preliminary stage. The changes of TLR genes and the expressions of important cytokines were investigated in this study in order to provide a reference for further study on the role of TLR genes in the exposure of TGEV-MSN.

IFN-α which belongs to type I IFN could activate and enhance the cytotoxicity of natural killer (NK) cells, promoting the maturation and activation of DCs [42]. As expected, DCs produced high levels of IFN-α after stimulation with inactivated TGEV vaccine with MSNs. DCs are also involved in the activation of NK cells by releasing IL-12, which enhances NK cytotoxicity and activates Th1 cells [43]. The inactivated TGEV vaccine with MSNs significantly increased the secretion of IL-12 in im-DCs. After treatment of DCs with inactivated TGEV vaccine with MSNs, we also observed that TNF-α and IL-1β were upregulated, which might be related to the inflammation activations of Th2 cells.

There are multiple factors that potentially influence the efficacy of vaccines. In this study, we focused on the effects of inactivated TGEV vaccine with MSNs on the phenotype and function of porcine DCs, and tried to explore the immune-enhancing mechanism of MSNs. However, it is well-known that the main pursued effect of vaccines used in pig production is individual direct protection. The protective effect of the vaccine is demonstrated by the decrease of pathogen shedding and the decrease of clinical symptoms in vaccinated animals. Vaccines can be used to offer clinical protection and control disease, which must be evaluated by specific experiments [44]. The protection test against the challenge with a virulent virus on the animal is vital in order to evaluate the vaccine. Furthermore, we should also pay attention to this point in future study.

## 5. Conclusions

In conclusion, we used biodegradable MSNs as adjuvant of the inactivated TGEV vaccine and explored the effects on the phenotype and function of porcine DCs. Results presented in this study indicated that MSNs could induce DC activation and maturation capacity. These data also demonstrated the mechanism by which inactivated TGEV vaccine with MSNs enhanced DC function during innate immunity.

## Figures and Tables

**Table 1:** Primers for expressions of TLRs, cytokines, and cytokine receptors in this study.

Genes	Accession Numbers in Genbank	Primers (5′-3′)	Amplicon Size (bp)
TLR-1	NM001031775.1	F: CCAGAGCTGCCAGAAGATTAG R:TCTACCACGTCACTCGATACT	114
TLR-2	NM213761.1	F: GGAGCCTTAGAAGTAGAGTTTGA R: AAGGGAACAGGGAACCAG	234
TLR-3	NM001097444.1	F: ATGCTCCGAAGGGTGG R: GGGTTTGCGTGTTTCC	111
TLR-4	NM001293316.1	F: CCACCTGTCAGATAAGCG R: CCTCACCCAGTCTTCGTC	102
TLR-5	NM001348771.1	F: CCACCAGGAGTCTTTCGC R: CGGCACTTAGTGAGGTGAAT	111
TLR-6	NM213760.2	F: ATCACCAGCCTCAAGCATTT R: TAGCCAGTTGTAAACACCCT	114
TLR-7	NM001097434.1	F: CCTTTCTGTCTCTCTGTGTCTTC R: CACCCTTCTCCCAACAGTATTT	81
TLR-8	NM214187.1	F: CTTTGATGATGACGCTGCTTTC R: GGTGTGTCACTCCTGCTATTC	99
TLR-9	NM213958.1	F: TTACTAGGGAGGTGGATGGTAG R: CCTTGCAGTTTGGCATGAAG	108
TLR-10	NM001030534.1	F: CACCACCACCTCTTCCATAAA R: AGAGCTTTCAGTGCAGGATAC	104
IL-1β	NM214055.1	F: ACCTGGACCTTGGTTCTC R: GGATTCTTCATCGGCTTC	124
IL-6	NM001252429.1	F: TTCAGTCCAGTCGCCTTCT R: GTGGCATCACCTTTGGCATCTTCTT	99
IL-12	NM214097.2	F: AGTGCCCTCAGTAAGAGTAAGA R: CTTGTGTTCCCACCCATCAA	86
TNF-α	NM214022.1	F: CCCTTGAGCATCAACCCT R: GCATTGGCATACCCACTCT	131
IFN-α	JQ839262.1	F: CATCCTGGCTGTGAGGAAATAC R: CAGGTTTGTGGAGGAAGAGAAG	118
CCR-7	NM_001001532.3	F: CGGTCATGTACTCCATCATCTG R: ATGGTCTTGAGCCTCTTGAAATA	91
β-actin	DQ452569.1	F: TTCCAGCCCTCCTTCCTG R: AGGTCCTTGCGGATGTCG	94

## Data Availability

The data used to support the findings of this study are available from the corresponding authors upon request.
